# Prevalence of Vitamin A Deficiency in South Asia: Causes, Outcomes, and Possible Remedies

**DOI:** 10.3329/jhpn.v31i4.19975

**Published:** 2013-12

**Authors:** Saeed Akhtar, Anwaar Ahmed, Muhammad Atif Randhawa, Sunethra Atukorala, Nimmathota Arlappa, Tariq Ismail, Zulfiqar Ali

**Affiliations:** ^1^Department of Food Science &Technology, Bahauddin Zakariya University, Multan, Pakistan; ^2^Department of Food Technology, PMAS-Arid Agriculture University, Rawalpindi 46300, Pakistan; ^3^National Institute of Food Science and Technology, University of Agriculture, Faisalabad, Pakistan; ^4^Department of Biochemistry & Molecular Biology, Faculty of Medicine, University of Colombo, Colombo, Sri Lanka; ^5^Division of Community Studies, National Institute of Nutrition, Indian Council of Medical Research, Tarnaka-Hyderabad 500 007, India; ^6^Department of Agriculture and Food Technology, Karakoram International University, Gilgit-Baltistan, Pakistan

**Keywords:** Blindness, Infections, Malnutrition, Vitamin A, South Asia

## Abstract

Vitamin A deficiency (VAD) has been recognized as a public-health issue in developing countries. Economic constraints, sociocultural limitations, insufficient dietary intake, and poor absorption leading to depleted vitamin A stores in the body have been regarded as potential determinants of the prevalence of VAD in South Asian developing countries. VAD is exacerbated by lack of education, poor sanitation, absence of new legislation and enforcement of existing food laws, and week monitoring and surveillance system. Several recent estimates confirmed higher morbidly and mortality rate among children and pregnant and non-pregnant women of childbearing age. Xerophthalmia is the leading cause of preventable childhood blindness with its earliest manifestations as night blindness and Bitot's spots, followed by blinding keratomalacia, all of which are the ocular manifestations of VAD. Children need additional vitamin A because they do not consume enough in their normal diet. There are three general ways for improving vitamin A status: supplementation, fortification, and dietary diversification. These approaches have not solved the problem in South Asian countries to the desired extent because of poor governmental support and supervision of vitamin A supplementation twice a year. An extensive review of the extant literature was carried out, and the data under various sections were identified by using a computerized bibliographic search via PubMed, Web of Science, and Google Scholar. All abstracts and full-text articles were examined, and the most relevant articles were selected for screening and inclusion in this review. Conclusively, high prevalence of VAD in South Asian developing countries leads to increased morbidity and mortality among infants, children, and pregnant women. Therefore, stern efforts are needed to address this issue of public-health significance at local and international level in lower- and middle-income countries of South Asia.

## INTRODUCTION

South Asia constitutes one-fifth of the world's population, and many of the nations have been severely affected by malnutrition. Vitamin A deficiency (VAD) has been established as a major determinant to deleteriously impact the health and economic status of populations in the lower-income South Asian countries, and it exists in poorer settings with economic deprivation. VAD is generally associated with decreased dietary intake of preformed vitamin A and its precursors, together with a high prevalence of infectious diseases, like measles, diarrhoea, and respiratory tract infections. Diets containing insufficient vitamin A lead to decreased serum vitamin A levels, resulting in various physiological implications, especially tissue development, metabolism, and resistance to infections. Severe VAD leads to xerophthalmia, the most common cause of preventable blindness among children (1,2).

Retinol (preformed vitamin A) exists in animal tissues, particularly liver and liver oil (cod liver oil), dairy products, and eggs; β-carotene and its precursor are derived from plant-based foods. Economic and sociocultural determinants lead the world community to rely on plant sources for meeting vitamin A requirements in the form of pro-vitamin, β-carotene, which is subsequently converted into retinol in the gut (3), and its activity is expressed in retinol activity equivalents (RAE): (1 RAE=1 µg retinol, 12 µg β-carotene).

Nearly 44-50% preschool children in South Asian regions were affected by severe VAD (2). Mortality owing to malnutrition and higher prevalence of VAD among neonates and children below 5 years of age in Bangladesh and India constituted one-third of the global mortality rate. Other estimates showed 1.02 billion people to be severely affected by micronutrient deficiencies globally, with vitamin A to be the most deficient nutrient in the body (4,5). Similar studies indicated that 85% of the total South Asian children with xerophthalmia reside in India (2). A significant increase in the magnitude of VAD among Indian women from 2001(5.9%) to 2011 (30.3%) was observed (6).

A study among pregnant women in five districts of Sri Lanka during 1988-1989 showed that 1.0% and 1.2% of women had night blindness and Bitot's spots respectively while 8.1% had low serum vitamin A (<20 µg/dL) during early pregnancy (7). Women (10%-15%) were also observed to be night blind during their third month of pregnancy. Approximately 31% children endured visual loss associated with childhood factors, over 75% of which was attributed to VAD (8). In fact, most children (>90%) who go blind from vitamin A deficiency die; so, they are not even counted in surveys of the prevalence of childhood blindness. Almost 5.7 million children below 5 years of age were identified as vitamin A-deficient in Pakistan (2). Increased risk of blindness was assessed in North West Frontier Province of Pakistan (NWFP) among children aged 6 years or less (9,10), and 16% of the anaemic children in primary schools in Karachi, Pakistan, were shown to be vitamin A-deficient. The risk of VAD in Pakistan is around 70% in pregnant and lactating mothers (11-14).

Lower vitamin A levels were reported in the breastmilk of mothers with VAD; therefore, nursing mothers with VAD transferred relatively lower concentration of vitamin A to the infants through breastfeeding. Pregnant women with VAD generally restrict supply of normal amount of retinol to foetus in late pregnancy (15,16). Malnutrition among under-five children is estimated to be 41% in Bangladesh. Many programmes, like vitamin A supplementation, to eliminate VAD in Bangladesh showed promise but the impact may take time to cover the entire population (17).

### Aetiology of vitamin A deficiency

Vitamin A is essentially required in the body to maintain visual system, sustain normal cellular differentiation, develop resistance against infections, and uphold epithelial integrity, red blood cell production, and reproduction. Primary vitamin A deficiency could be attributed to prolonged deprivation of vitamin A-rich foods and is further depleted by diarrhoea, measles, and respiratory infections. VAD also prevails among populations whose diets are lacking in animal products (3). This is almost always the case because a young child cannot possibly consume sufficient dietary sources of beta-carotene to satisfy their vitamin A needs from vegetables and grains alone.

Children and pregnant women are more likely to suffer from VAD. Infants fed no or little breastmilk in early life are increasingly susceptible to various maladies. The colostrum discarded usually results in failure to maintain normal vitamin A levels among infants. One recent study confirmed the role of high dose of vitamin A supplementation to significantly reduce progressive hearing loss associated with purulent ear infections in early childhood (18).

Poor bioavailability plays a predominant role in the development of VAD among communities that mainly rely on plant-based foods. Moreover, significantly higher prevalence of VAD in South Asian developing countries may overwhelmingly disintegrate the health and economic infrastructure of these societies. It is imperative that immediate action be taken to meet the VAD challenge.

### Vitamin A deficiency in South Asian perspective

Worldwide, undernutrition leads to one-third of the total deaths among children. The highest child malnutrition and mortality is reported in South Asia, and data indicate that 178 million of the children below 5 years of age go stunted while global estimate of wasting is ~55 million children, of whom 19 million were severely wasted. Other studies demonstrated that approximately 48% of under-five children in India alone were stunted, followed by 43% in Bangladesh and 37% in Pakistan (19-22).

Most vitamin A-deficient children live in South-East Asia where 91.5 million preschool children had serum retinol concentrations <0.70 μmol/L, i.e. <20 μg/dL. Moreover, night blindness in preschool children was the highest in South-East Asia (82.4%) compared to very low in Europe (1%) and almost nil (0%) in America (2). The extent of prevalence of VAD among children in South Asian developing countries is presented in Table 1. These data clearly illustrate the gravity of VAD prevalence as a public-health problem in South Asia. Numerous trials conducted in South Asia reported a significant reduction in mortality (21%) in the first six months of life among neonates following vitamin A supplementation (23), and 40% reduction in maternal mortality was witnessed following routine dietary supplementation with vitamin A during pregnancy (24).

### Vitamin A deficiency in India

India has the highest prevalence of clinical and subclinical VAD among South Asian countries; 62% of preschool children were reported to be deficient in vitamin A. These dramatic results suggested high mortality rate, leading to an annual 330,000 child deaths. Estimates confirmed 31% to 57% preschool children to be the victims of subclinical VAD. Women of childbearing age excessively suffered from night blindness, with 5% pregnant women manifesting subclinical VAD. Among these 5%, about 12% were severely affected with night blindness during pregnancy (25,26). International Institute for Population Sciences, India, confirmed higher prevalence of night blindness among pregnant women, with higher percentage among rural population compared to urban folks (rural 13.7%, urban 6.4%) (27).

India, being a very vast country, represents a variety of sociocultural and economic settings. This corresponds to the diversified magnitude of VAD prevalence in the region, e.g. the number of children with vision problem significantly varied from region to region. Despite tremendous efforts directed at all levels to curtail VAD in India, the prevalence of subclinical VAD still exists as one of the highest in the world. High magnitude of productivity loss owing to higher prevalence of malnutrition in India has been reported (28). Consequently, Government of India recommended vitamin A supplementation programme with every six months interval starting at an age of 9 months among infants until they reach five years of age. Another strategy to combat VAD is applied in the form of supplementation of a massive dose to prevent nutritional blindness among preschool children in India for the last three decades; however, the coverage remains a potential barrier to achieving the goal.

### Vitamin A deficiency in Pakistan

Maternal and child health conditions are significantly precarious in Pakistan, and the country faces elevated child mortality rates annually due to diarrhoea and pneumonia infections. This situation depicts the extent of healthcare facilities available to the vulnerable population groups in Pakistan. VAD in Pakistan is considerably pervasive, thus declaring Pakistan as a country with “severe subclinical deficiency of vitamin A.”

Children suffering from measles were particularly reported to suffer from VAD, which was subsequently controlled on dispensing one to two mega-doses of vitamin A. Approximately 53% avoidable cases of blindness, of which 58% could be preventable, were identified in a local school for blind and deaf children in Karachi (11). Low serum retinol concentration among pregnant women with night blindness in Karachi was also detected, suggestive of higher VAD prevalence in periurbun areas of Karachi. These visual symptoms were locally named ‘*chhaya and shafkoor*’ meaning shadow and inability to see at night (29).

**Table 1.** Prevalence of vitamin A deficiency (VAD) among children in South Asia (%)

Country	Children <6 years		
	No. of deaths perceived	Subclinical VAD (%)	Clinical VAD (%)
Afghanistan	50,000	53	-
Bangladesh	28,000	28	0.7
Bhutan	600	32	0.7
India	3,30,000	57	0.7
Nepal	6,900	33	1
Pakistan	56,000	35	-
South Asia region	4,71,500	-	-
World total	11,50,000	-	-

Derived from UNICEF 2003 (19): UNICEF and MI 2004 (26): WHO 2000 (70)

Clinical cases of VAD, especially among children below 6 years of age, were excessively reported in major provinces of Pakistan (30). Khyber Pakhtunkhwa (KPK) and Federally Administrated Tribal Area (FATA) were shown to be predisposed to VAD. High risks of blinding xerophthalmia associated with systemic illness in various community settings and age-groups were identified in Pakistan (10). Restricted access of communities to foods rich in vitamin A and holding low socioeconomic status are normally reflected as VAD (31). No holistic study to estimate the real burden of VAD has yet been carried out in Pakistan. However, the recent National Nutrition Survey (6) with 6,925 males and 912 females, covering all four provinces of Pakistan (Table 2) portrays the level of VAD in the country. The data so obtained simply presented VAD among pregnant and non-pregnant women and are seemingly meagre to reflect the true picture and the gravity of the prevalence of VAD in the region. More concerted efforts need to be directed to planning a complete countrywide study, covering large populations from all groups.

### Vitamin A deficiency in Sri Lanka

VAD has been identified as a problem of public-health significance in Sri Lanka. National survey with clinical and laboratory assessment was conducted by the Medical Research Institute (MRI) in 1995-1996, covering 2,869 children aged 6-71 months to assess the magnitude of the problem among preschool children (32). Following this survey, the national policy was reformulated, and a VAD control programme was developed. The focus of this programme was to improve the vitamin A status of preschool and primary school children, pregnant and lactating mothers, and displaced persons and refugees.

The prevalence of VAD was re-assessed in Sri Lanka by MRI in a cross-sectional survey among preschool children during 2005-2006, suggesting severe VAD prevalence in the region. The proportion of children with VAD was significantly higher in the presence of respiratory tract infections during the two weeks prior to the survey. Based on these findings, recommendations were presented to repeat supplementation every six months until five years of age and to promote increased consumption of vitamin A-rich foods (33).

**Table 2.** Vitamin A deficiency in non-pregnant and pregnant mothers in Pakistan

Level of deficiency		Total	Residence		Province/Region						
			Urban	Rural	Punjab	Sindh	KPK	Baluchistan	FATA	AJK	Gilgit
Severe (<0.35 μmol/L)	NPM	17.8	10.1	21	19.2	10.3	38	20.3	48.2	0.5	8.5
	PM	19.9	15.6	21.5	20.5	16.5	47.7	26.7	0	0	20.9
Mild (0.35-0.70 μmol/L)	NPM	25.4	24.2	25.9	24.3	25.9	33.7	29.9	35	12	30.5
	PM	28.8	25.8	30	26	33	37.5	35.8	0	30.6	23.6
Non-deficient (>0.70 μmol/L)	NPM	56.9	65.7	53.1	56.6	63.8	28.3	49.8	16.8	87.5	61
	PM	1.2	58.6	48.4	53.5	50.5	14.8	37.5	0	69.4	55.5
Subject (No.)	NPM	6,925	2,686	4,239	3,502	1,819	434	435	57	394	284
	PM	912	329	583	497	285	30	36	0	42	22

AJK=Azad Jammu Kashmir;

FATA=Federally Administered Tribal Areas;

KPK=Khyber Pakhtunkhwa;

NPM=Non-pregnant mothers;

PM=Pregnant mothers;

Derived from NNS 2011 (6)

The food-based approaches followed in Sri Lanka to control VAD included dietary diversification, cultivation of vitamin A-rich crops in home gardens, promotion of breastfeeding, and enhancing fat and vitamin A-rich foods in complementary feeding. In addition, the provision of vitamin A (100,000 IU) orally to preschool children at 9 months (with measles immunization) and at 18 months (200,000 IU) to postpartum mothers within 4 weeks after delivery, and a single dose (100,000 IU) orally to school children in school years 1, 4, and 7 was created with assistance from UNICEF and the Micronutrient Initiative (34).

Subclinical VAD still remains a public-health problem in Sri Lanka, despite the availability of a supplementation programme for children and pregnant and lactating women. Sagacious and practicable approaches are needed to appraise the impact of the supplementation programme in primary healthcare settings in relation to cost and benefit.

### Vitamin A deficiency in Bangladesh

Prevalence of subclinical VAD in adolescent boys remained relatively lower than other population groups in Bangladesh. A representative study illustrated approximately 1.5% of 381 school children (aged 11-16 years) of Dhaka to suffer from subclinical VAD, i.e. serum retinol <0.70 µmol/L. Another study reported that 51% pregnant women had deficit in diets to meet RDA for vitamin A, and 18.5% manifested VAD (serum retinol <0.70 µmol/L), suggesting VAD to be highly prevalent among pregnant women in rural Bangladesh. The indicators identified for this nutritional status were dietary habits and gestational age (35,36).

Several reports confirmed dramatic reduction in clinical or subclinical cases of night blindness among preschool children. Significantly positive results were achieved on launching intervention programmes of vitamin A supplementation in 1973 (37-39).

Supplementation is the most widely-adopted approach to preventing VAD in Bangladesh, typically focusing children and pregnant women. Vaccination programmes were exploited for vitamin A supplementation; children aged 9-11 months received vitamin A capsules (100,000 IU) at the time of measles vaccination whereas children aged 12-59 months were given 200,000 IU every six months. Prevalence of VAD in Bangladesh and associated visual implications among various age-groups have been presented in Table 3. Literacy level among mothers appeared to be a significant determinant of vitamin A supplementation. Mothers with primary education had greater inclination for vitamin A supplementation compared to those having no education or incomplete primary education (39).

Estimating magnitude of the prevalence of VAD among various population groups and its elimination in Bangladesh has been a major concern of the international organizations. Evidently, VAD has been more seriously addressed in Bangladesh compared to the adjoining countries, such as India, Pakistan, Afghanistan, and Nepal. Several intervention programmes initiated in the past resulted in significant reduction in night blindness cases among children in Bangladesh; however, serum retinol levels as a measure to assess VAD indicated that the VAD is considerably pervasive and is a public-health problem. Restricted dietary intake has been an established factor of VAD among different groups. Alleviation of the problem of VAD in Bangladesh requires a long-term pragmatic approach covering all possible sectors. Government priority to invest in VAD elimination is the most significant one among these approaches.

**Table 3.** Prevalence of vitamin A deficiency in Bangladeshi population

Deficiency	Population-size (Number)	Gender	Age	Percentage
Night blindness	27,574	Male/Female	6-59 months	0.67
Bitot's spot	27,574	Male/Female	6-59 months	0.25
Corneal xerosis	27,574	Male/Female	6-59 months	0.01
Xerophthalmia	27,574	Male/Female	6-59 months	0.1
Nightblindness	6,827	F	15-49 years	2
Nightblindness	2,461	F*	15-49 years	2.7
Nightblindness	14,381	F†	15-49 years	2.4

F=Non-lactating non-pregnant;

F*=Pregnant women;

F†=Lactating women; Derived from HKI 1999 (37): Anon 2004 (34)

## VITAMIN A DEFICIENCY: RISKS AND OPPORTUNITIES

### Health implications of vitamin A deficiency

Among many physiological roles of vitamin A, normal functioning of the visual system, maintaining cell function to promote growth, producing red blood cell (RBCs), developing immunity, and reproduction are all significantly important. VAD generally results in night blindness, severe anaemia, wasting, reproductive and infectious morbidity, and increased risk of mortality (40-42). Visual implications due to VAD among vulnerable groups are exceedingly pervasive among low-income countries of South Asia (4,5,43).

Various reports validated the mechanistic weakening of local barriers to infection by a substantial reduction in lymphocyte response (44) that led to reduced secretory IgA (Immunoglobulin A) levels in mucous membranes during VAD (45). This confirmed more susceptibility of vitamin A-deficient women to illness from both infectious (40) and non-infectious diseases (46,47).

A recent estimate showed 1,000 children to be infected everyday with HIV transmitted from mothers worldwide. This situation amplifies the need for vitamin A supplementation among HIV-infected pregnant women, speculating that vitamin A supplements to breastfeeding women might reduce the likelihood of their infants being infected with HIV. However, the available body of literature does not confirm the use of vitamin A supplementation in HIV-infected pregnant or breastfeeding women to reduce mother-to-child transmission of HIV (48).

### Strategies to controlling vitamin A deficiency

Despite a number of other trials to control VAD, three viable approaches have been still in place to achieving the goal of elimination of VAD, i.e. dietary diversification, supplementation, and fortification. These strategies have been attempted depending upon the gravity of the VAD in various sociocultural settings. These major strategies, along with other approaches, are discussed in the perspective of South Asian developing countries.

### Dietary diversification

Dietary diversification has shown some advantages over other interventions to control VAD. This approach seems sustainable and calls for no external support. It holds the ability to concurrently cover multiple micronutrient deficiencies. This approach, if supported with a nutrition education programme, may be more effective in the developing countries. Likewise, VAD problem may be addressed exploiting food-based strategies as a permanent solution of the problem in developing countries.

Consumption of a variety of vitamin A-rich foods gained popularity in India to combat micronutrient deficiencies, and dietary diversification was promoted to ensure nutrition security through health and nutrition education for sustainable and long-term results to control VAD (49,50). Little efforts have been directed in Pakistan to promoting dietary diversification. Most recently, a campaign has been launched to promote kitchen gardening in the province of Punjab through the supply of seasonal vegetable seeds at subsidized prices on demand, with counselling. Apparently, the outcome of this approach does not seem to have positive impact on the VAD prevalence in Pakistan. Similarly, dietary diversification is used as a sustainable method for improving vitamin A status in Sri Lanka where subclinical VAD continues to be a health concern. Nutrition education plays a key role in achieving dietary diversification. Educational intervention resulted in a significant increase in knowledge and consumption of local vitamin A-rich foods.

Home gardening as a food-based strategy reflected promising results in Bangladesh; however, it seemed much demanding to continue these practices because of inadequate counselling and education on nutrition. Recent investigations on home gardening as a strategy in South Africa revealed the efficacy of this approach to controlling VAD to a substantial level (51,52).

### Supplementation

Supplementation is regarded as the most effective strategy and is widely practised to replenish VAD in low-income countries. Dispensing potent supplements (200,000 IU of vitamin A) in a periodical manner to under-5 children or 100 000 IU of vitamin A given to infants aged 6-11 months has shown promise to control VAD in many developing countries in South Asia (53). Side-effects of mega-doses (200,000 IU) of vitamin A supplements, i.e. 500 times of the RDA (400 IU) for children, have been extensively debated as large doses may be more harmful than beneficial (54,55).

National vitamin A supplementation programme initiated in Sri Lanka indicated rational success (56). Absenteeism from school as a marker to gauge efficacy of supplementation with a mega-dose (200,000 IU) of vitamin A in Sri Lanka validated that supplemented children lost fewer number of school days due to illness compared to the placebo group (57). Data pertaining to coverage of vitamin A supplementation in Bangladesh exhibited high coverage among children aged 9-11 and 12-59 months (58). However, this does not imply that the targets to overcome VAD are achieved; more efforts are needed to address the issue, using a combination of approaches, like production, regulation, and consumption of vitamin A-rich foods.

Vitamin A supplementation in the form of vitamin A drops has been in progress since 1999 in Pakistan, and a dose of 20,000 IU of vitamin A is dispensed at suitable intervals. This dose is reduced to half in infants aged less than one year (8 kg body-weight). Mega-doses of vitamin A to neonates after six months of age have been recommended to control higher mortality rate among infants. A considerable reduction (~24%) in VAD among infants and children was, thus, seen in Pakistan on the administration of high doses of vitamin A (29,59,60).

Estimates show an overall reduction in mortality by 23% in India through vitamin A supplementation (61); however, these claims were already contradicted in another study where the effect of vitamin A supplementation on mortality was indicated to be much low in India (62,63).

Conclusively, supplementation of vitamin A as an approach to eradicating VAD in South Asian developing countries is beneficial provided the programmes are well-structured and appropriate monitoring and surveillance practices are followed. Data pertaining to vitamin A supplementation coverage rates among children aged 6-59 months in South Asian developing countries show higher coverage in Pakistan, Nepal, and Afghanistan, followed by Bangladesh.

### Fortification

Fortification of a staple food with vitamin A is now well-recognized as a viable strategy to increasing the dietary intake of vitamin A. Developing economies have learnt lessons from the experience of Central and South America where VAD control was effectively achieved through fortification of sugar 30 years back. Therefore, bright prospects are existent for vitamin A fortification of foods to considerably reduce VAD in the developing countries (64).

Fortification of vegetable oil with vitamin A is now a well-established approach to achieving desired results. Fortified vegetable oils do not exhibit any change or safety issue for higher intakes provided the recommended levels of fortification are followed. Mandatory fortification of cooking oils with vitamin A has been a focus of the developing societies to combat VAD, e.g. all oils must be fortified at 33 IU/g and 20 IU/g in Pakistan and India respectively (65). Similarly, 300,000 metric tonnes of edible oil is being fortified in Bangladesh as a strategy for controlling VAD among children and women. The project is much cost-effective and will be beneficial for 90 million children and women in Bangladesh (66).

Vegetable oil fortification with vitamin A is poorly managed in Pakistan because of the absence of any precise surveillance and monitoring system, and virtually, a few of the edible oil processors fortify the oil in accordance with the law. This is evident from the presence of considerably varying vitamin A content in fortified oil/ghee brands in Pakistan (67).

Fortification of cereal-based products, like cookies, cakes, and pastries containing fat up to 20-30% with vitamin A has been extensively explored for VAD control in developing countries. Some studies in Pakistan validated the efficacy of vitamin A-fortified cookies, and promising results were achieved for baking and storage stability of the product, thus suggesting fortification of cookies with retinyl acetate (14,68,69). However, the selection and levels of appropriate fortificant need to be particularly considered for fortification, e.g. appropriate levels and bioavailability are two substantial factors that can contribute to improving nutrient status among populations with very little risk of adverse effects (70).

Controlling VAD situation in South Asian developing countries appears to be a demanding task, and obviously, a single strategy does not seem like working effectively. A multipronged approach needs to be in place, including fortification as one of the most potential long-term approach for all target groups. Mandatory fortification of vegetable oils in South Asian developing countries needs to be expanded and strictly enforced.

### Other strategies to controlling VAD in South Asian countries

#### Removal of socioeconomic barriers

Many of the South Asian developing countries face financial constraints, resulting in limited access of the population to animal products containing higher amount of vitamin A in the form of retinol. Rearing of animals and promoting poultry, dairy cows, and/or fish are some of the indirect approaches to minimizing VAD among rural populations of South Asian countries. Plant sources of vitamin A include β-carotene (pro-vitamin A) mainly derived from fruits and vegetables with relatively low bioavailability compared to retinol. Alleviating VAD through more vegetable cultivation and production of fruits among communities with lower socioeconomic status has been strongly suggested by many researchers. These practices offer the growers a direct access to vital and expensive nutrients which otherwise are hard to afford. This strategy is being followed in India and Bangladesh. Postharvest handling and cooking practices are two important determinants for loss of vitamin A. Availability of (pro) vitamin A can be substantially assured through suitable storage and food processing (71).

#### Nutrition education

Awareness of the harmful impact of VAD may greatly curtail its prevalence in developing countries. For example, educated mothers and community members are able to detect signs of vitamin A deficiency at the earlier stages, enabling them to control the disease timely. Knowledge on the beneficial effect of consumption of micronutrient-rich foods, like green leafy vegetables, poultry meat, cereal, and dairy products, may lessen the VAD risk to a considerable extent.

Educating mothers on VAD in low-income South Asian developing societies holds a potential to reduce the gravity of VAD in these regions, e.g. children aged less than five years did not receive vitamin A supplementation owing to low maternal education in Bangladesh (72). Only 24% mothers were shown to have some rudimentary knowledge about vitamin A, of whom 41.6% were from cities. About 78.1% of mothers were unaware of health implications of VAD in Pakistan (10,15).

#### Sanitation and hygiene

Nearly 4.5 billion people are prone to VAD owing to intestinal parasite infections worldwide. Round worm, typically *Ascaris lumbricoides*, are the most implicated means of causing intestinal parasitical problems in the developing world (73,74) while Trichuriasis was associated with a low vitamin A status among adolescent school girls of low socioeconomic status in Sri Lanka (75). Evidently, foodborne diseases have gravely distressed the populations in the developing countries in terms of health and economics (76). Mould proliferation in stored foods due to high humidity and temperature in these countries exacerbates food safety issues (77). Environmental sanitation, safe water supply, proper hygiene and food safety, regular deworming, and immunization practices against diphtheria, pertussis, tetanus, typhoid, and cholera can substantially reduce the risk of VAD in South Asian developing countries. Therefore, practising proper sanitation and hygiene in addition to antihelminthic therapy might have a synergistic effect to eliminate poorer economies.

### Conclusions

A substantial number of children and pregnant/lactating women are the victims of vitamin A deficiency in South Asian developing countries due to poverty and allied socioeconomic constraints. Insufficient dietary intake of vitamin A is a predominant cause of developing VAD in India, Pakistan, Bangladesh, and Sri Lanka. VAD normally leads to the loss of health and productivity as well. Several approaches to controlling VAD in South Asian countries have been attempted, including dietary diversification, supplementation, and fortification. Mega-doses of vitamin A delivered to infants and children in these countries showed encouraging results; however, dispensing mega-doses to infants initiated a debate on vitamin toxicity. Lessons learnt from the developed economies for exploiting food fortification as a viable strategy to controlling VAD were seriously followed in the form of fortified cereal-based baked products and vegetable oils. Dietary diversification as a tool to combat VAD has been strongly recommended, and especially India's and Bangladesh's experiences yielded potential benefits of such programmes. Understanding how to avoid the development of prevalence of VAD is a key approach to minimizing VAD in South Asian developing societies. Health consequences of VAD are much damaging and deleteriously impact all segments of population, especially pregnant women and children. Poorer economies have to pay huge cost in terms of health and productivity. New paradigms clearly define VAD as the most central issue to be addressed on emergent grounds to sustain health and wellbeing of population residing in developing countries.

## ACKNOWLEDGEMENTS

We are highly grateful to Dr. Sommer Alfred, Dean Emeritus, Johns Hopkins Bloomberg School of Public Health, Professor of Ophthalmology, Epidemiology & International Health, Johns Hopkins School of Medicine, USA, for his significant technical support and constructive criticism to improve the manuscript.
